# Comparison of the muscle fascicle length between resistance-trained and untrained individuals: cross-sectional observation

**DOI:** 10.1186/s40064-015-1133-1

**Published:** 2015-07-11

**Authors:** Atsuki Fukutani, Toshiyuki Kurihara

**Affiliations:** Research Organization of Science and Technology, Ritsumeikan University, 1-1-1 Noji-higashi, Kusatsu, Shiga 525-8577 Japan; Japan Society for the Promotion of Science, 5-3-1 Kojimachi, Chiyoda-ku, Tokyo, 102-8472 Japan; Faculty of Sport and Health Science, Ritsumeikan University, 1-1-1, Noji-higashi, Kusatsu, Shiga 525-8577 Japan

**Keywords:** Ultrasonography, Pennation angle, Muscle thickness, Medial gastrocnemius, Vastus lateralis

## Abstract

**Background:**

Muscle plasticity is an important topic in the fields of sport science and physical therapy. The purpose of this study was to examine whether muscle fascicle length increases with resistance training through a comparison between resistance-trained and untrained individuals. If a hypothesis that fascicle length increases by resistance training is true, fascicle length should be longer in the resistance-trained individuals than in the untrained individuals.

**Findings:**

Sixteen individuals (age, 21.3 ± 3.3 years; height, 1.74 ± 0.05 m; body mass, 83.6 ± 9.0 kg), who were either body builders or rugby players, were recruited as the training group, and 11 individuals (age, 20.3 ± 2.1 years; height, 1.70 ± 0.06 m; body mass, 54.0 ± 4.7 kg), who did not have regular resistance training experiences, were recruited as the control group. Fascicle length, pennation angle, and muscle thickness of the vastus lateralis and medial gastrocnemius were measured from ultrasonographic images. We found that the muscle thickness and pennation angles in the training group were significantly larger than those in the control group (*p* < 0.05). However, fascicle length did not significantly differ between the two groups (*p* > 0.05).

**Conclusions:**

These results indicate that fascicle length is not associated with muscle size, suggesting that fascicle length would not increase with resistance training.

## Findings

### Background

Muscle morphology affects the characteristics of muscle contraction. The cross-sectional area of a muscle determines the muscle force (Ikai and Fukunaga 1968) [in the case of pennation muscle, physiological cross-sectional area is a better index than anatomical cross-sectional area (Morse et al. 2008)] and the length of muscle fibres have a strong influence on the shortening velocity of the muscle fibres (Bodine et al. 1982). Thus, in the fields of sport science and physical therapy, clarification regarding the morphological plasticity of muscle is important for enhancing the performance of human movement.

To date, it is widely accepted that the cross-sectional area of a muscle increases with resistance training (Häkkinen et al. 2001; Moore et al. 2012). In addition, the pennation angle also increases in association with muscle hypertrophy (Aagaard et al. 2011; Blazevich and Giorgi 2001). However, whether or not the length of the muscle fibre (measured as fascicle length) increases with resistance training has been controversial (Ema et al. 2013; Erskine et al. 2010; McMahon et al. 2014; Potier et al. 2009; Seynnes et al. 2007). For example, Potier et al. (2009) reported that the fascicle length of the biceps femoris increased after 8 weeks of resistance training. In addition, Seynnes et al. (2007) reported only 10 days training increased fascicle length in vastus lateralis. On the other hand, Erskine et al. (2010) could not confirm the increase in fascicle length of the vastus lateralis (VL) after 9 weeks of resistance training. These conflicting results might be caused by the relatively short duration of the resistance training interventions. Considering the fact that it would take about 12 weeks for significant architectural adaptation of the muscle to resistance training (Folland and Williams 2007), it is better to adopt a much longer intervention period compared to previous studies, or to compare highly-trained and untrained individuals, to determine whether fascicle length increases with resistance training. Therefore, the purpose of this study was to examine whether resistance training increases fascicle length by using cross-sectional study, that is, comparing resistance-trained and untrained individuals. If the hypothesis that fascicle length increases by resistance training is true, fascicle length should be longer in the highly-trained individuals than in the untrained individuals.

## Methods

### General concept

The fascicle length, pennation angle, and the thickness of VL and medial gastrocnemius (MG) were measured by ultrasonography (SSD-3500, ALOKA, Tokyo, Japan) with a linear array probe (7.5 MHz, UST-5710; Aloka, Tokyo, Japan) in highly-trained and untrained individuals. These parameters were measured at three joint angles because fascicle length-joint angle relationship may be different between two groups. In this study, all the measurements were conducted on the right side of the body.

### Subjects

Sixteen individuals (age, 21.3 ± 3.3 years; height, 1.74 ± 0.05 m; body mass, 83.6 ± 9.0 kg), who were either body builders or rugby players, were recruited as the training group and 11 individuals with no experience in regular resistance training (age, 20.3 ± 2.1 years; height, 1.70 ± 0.06 m; body mass, 54.0 ± 4.7 kg) were recruited as the control group. Subjects in the training group had attended the national level sport festivals. Each subject was explained the purpose and risks of the study, and written informed consent was obtained from all subjects.

### Measurements

Ultrasonographic images of VL were obtained from the mid-portion of the upper leg (at 50% of the length from the greater trochanter to the knee crease), and those of MG were obtained from the mid-portion of lower leg (at 30% of the length from the knee crease to the lateral malleolus). For the VL measurements, the subjects sat on the chair with the hip joint flexed at 90° (Anatomical position was defined as 0°). An ultrasonographic probe was set on the mid-portion of the upper leg with a double-faced tape, surgical tape and an underwrap. Ultrasonographic images of VL were recorded from three joint angle conditions. Knee joint angle was set at 0° (full extension), 45°, and 90° while hip joint angle was constant (90°). The knee joint angle was confirmed by the specially-designed angle measurement device (Z813-153A, FRIGZ, Tokyo, Japan). The images were then recorded by a personal digital video camera (DCR-HC90-NTSC, SONY, Tokyo, Japan). For the MG measurements, the subjects sat on the force-recording machine (Biodex, SAKAImed, Tokyo, Japan) with the hip joint in 70° flexion and the knee joint in 0° flexion. The ultrasonographic probe was set on the mid-portion of the lower leg by using a double-faced tape, surgical tape and an underwrap. Ultrasonographic images of the MG were recorded from three joint angle conditions. The ankle joint angle was set at 15° dorsiflexion, 0° plantar flexion, and 25° plantar flexion while knee joint angle was fixed at 0°. The ankle joint angle was controlled by the force-recording machine (Biodex, SAKAImed, Tokyo, Japan). The images were recorded on a personal computer in a similar fashion with VL measurements. In addition, we measured the length of the upper (from the greater trochanter to knee crease) and lower (from the knee crease to lateral malleolus) legs by tape measure to confirm that the difference in the muscle architecture was caused by muscle hypertrophy and not by the bone length.

### Data analyses

Ultrasonographic images were converted to the Audio Video Interleaved (AVI) files. Image analysis software (Image J, 1.47v, National Institute of Health, Maryland, USA) was used to measure the fascicle length, pennation angle and muscle thickness. Fascicle length was defined as the distance between the intersection composed of the superficial aponeurosis and fascicle and the intersection composed of the deep aponeurosis and the fascicle. Pennation angle was defined as the internal angle composed of the fascicle and deep aponeurosis. Muscle thickness was defined as the vertical line from the superficial aponeurosis to deep aponeurosis at the centre of the ultrasonographic images.

### Statistical analyses

Descriptive data are presented as mean ± SD. A two-way analysis of variance (ANOVA) (group × joint angle) was used to test the effects of the group and joint angle on the fascicle length, pennation angle, and muscle thickness of VL and MG. If the interaction or main effects were significant, additional one-way ANOVA with post hoc tests (unpaired *t* test for groups or paired *t* test with a Bonferroni correction for joint angle) were used. The level of statistical significance was set at *p* < 0.05. Effect size was calculated as Cohen’s *d* or partial *η*^*2*^. Statistical analyses were performed using SPSS version 20 software (IBM, Tokyo, Japan).

## Results

**Figure 1:**
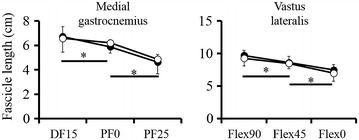
Fascicle length of the medial gastrocnemius and vastus lateralis. The *filled circles* represent the training group; the *empty circles* represent the control group. *Significant difference among the joint angles (*p* < 0.05).

For the fascicle length, two-way ANOVA revealed that no significant interactions [VL: *F* = 0.613, *p* = 0.532, effect size (partial *η*^*2*^) = 0.025; MG: *F* = 0.791, *p* = 0.459, effect size (partial *η*^*2*^) = 0.036] and no significant main effects between two groups [VL: *F* = 1.042, *p* = 0.318, effect size (partial *η*^*2*^) = 0.042; MG: *F* = 0.219, *p* = 0.644, effect size (partial *η*^*2*^) = 0.010] were found in both VL and MG. On the other hand, the significant main effects among joint angles were found in both VL and MG [VL: *F* = 46.131, *p* < 0.001, effect size (partial *η*^*2*^) = 0.658; MG: *F* = 117.010, *p* < 0.001, effect size (partial *η*^*2*^) = 0.848]. Fascicle length was significantly shorter with decrease in knee flexion angle for the VL and with increase in ankle plantar flexion angle for the MG in both groups (*p* < 0.001) (Figure 1).

**Figure 2:**
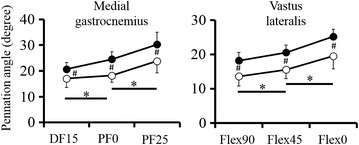
Pennation angle of the medial gastrocnemius and vastus lateralis. The *filled circles* represent the training group; the *empty circles* represent the control group. *Significant difference among the joint angles (*p* < 0.05). ^#^Significant difference between the two groups (*p* < 0.05).

For the pennation angle, two-way ANOVA revealed that no significant interactions were found in both VL and MG [VL: *F* = 0.229, *p* = 0.796, effect size (partial *η*^*2*^) = 0.009; MG: *F* = 3.370, *p* = 0.051, effect size (partial *η*^*2*^) = 0.138]. However, significant main effects were found between two groups [VL: *F* = 56.581, *p* < 0.001, effect size (partial *η*^*2*^) = 0.702; MG: *F* = 16.056, *p* < 0.001, effect size (partial *η*^*2*^) = 0.433] and among joint angles [VL: *F* = 53.729, *p* < 0.001, effect size (partial *η*^*2*^) = 0.691; MG: *F* = 73.004、*p* < 0.001, effect size (partial *η*^*2*^) = 0.777]. The pennation angle increased significantly with knee joint extended for VL and with ankle join flexed for MG in both groups (*p* = 0.001–0.002) (Figure 2).

**Figure 3:**
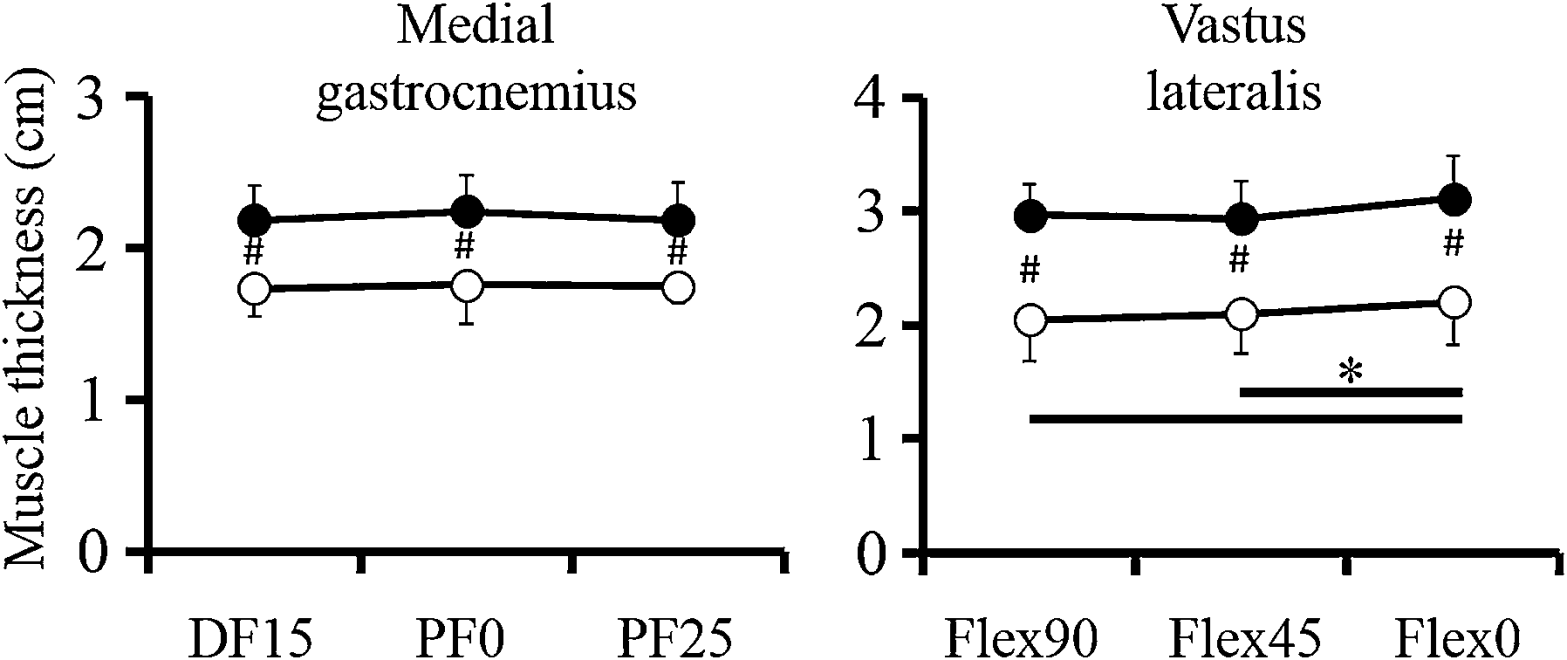
Muscle thickness of the medial gastrocnemius and vastus lateralis. The *filled circles* represent the training group; the *empty circles* represent the control group. *Significant difference among the joint angles (*p* < 0.05). ^#^Significant difference between the two groups (*p* < 0.05).

For muscle thickness, two-way ANOVA showed no significant interactions [VL: *F* = 0.344, *p* = 0.711, effect size (partial *η*^*2*^) = 0.014; MG: *F* = 2.297, *p* = 0.126, partial *η*^*2*^ = 0.152]. However, significant main effects between two groups were found [VL: *F* = 57.531, *p* < 0.001, effect size (partial *η*^*2*^) = 0.697; MG: *F* = 35.649, *p* < 0.001, effect size (partial *η*^*2*^) = 0.603]. In addition, significant difference was found among the joint angles for VL [*F* = 4.699, *p* = 0.013, effect size (partial *η*^*2*^) = 0.158]. Additional analyses revealed that muscle thickness was significantly larger in 0° compared with 45° (*p* = 0.018) and 90° (*p* = 0.022) (Figure 3).

There was no significant difference in upper [training: 40.0 ± 1.4 cm, control: 39.5 ± 2.4 cm, *p* = 0.569, effect size (*d*) = 0.254] and lower [training: 39.7 ± 1.9 cm, control: 39.0 ± 2.7 cm, *p* = 0.313, effect size (*d*) = 0.334] leg length between two groups.

## Discussion

In this study, we compared the fascicle length between resistance-trained and untrained individuals. It was found that fascicle length was not different between the two groups, although muscle thickness which is an index of muscle hypertrophy was significantly different. These results indicate that fascicle length is not associated with muscle size. Thus, our results did not support the aforementioned hypothesis that fascicle length increases by resistance training, in other words, muscle hypertrophy is not necessarily accompanied with increase in fascicle length.

As described in the introduction, there are conflict results regarding the fascicle length plasticity by resistance training. For example, previous studies have reported that fascicle length increased with several weeks of resistance training (Baroni et al. 2013; Blazevich et al. 2007a; Potier et al. 2009) although some other studies reported no increase in fascicle length (Blazevich et al. 2007b; Ema et al. 2013; Erskine et al. 2010). Thus, not only the precise mechanism but also whether fascicle length increases or not by resistance training is unclear at present. We confirmed that fascicle length was similar between groups which has large difference in muscle size. Thus, we think that the plasticity of fascicle length is low although some studies reported increase in fascicle length only several weeks of resistance training (Baroni et al. 2013; Blazevich et al. 2007a; Potier et al. 2009; Seynnes et al. 2007). If the fascicle length increases by several times of resistance training like Seynnes et al. 2007, resistance trained group in this study should have longer fascicle length because they have performed hundreds of resistance training. Thus, it is reasonable that fascicle length does not necessarily increase by resistance training.

We agree that fascicle length (i.e. sarcomere number in series) could increase in some cases. For example, Koh and Herzog (1998) reported that the sarcomere number in series for tibialis anterior increased after removing the extensor retinaculum of the ankle because a larger excursion of the muscle fibres occurred compared to that before removal of the extensor retinaculum. In addition, Boakes et al. (2007) confirmed that after surgical femoral lengthening, fascicle length of the VL increased dramatically (from 90 to 190 mm) and reported that this large increase in fascicle length was caused by an increase in the sarcomere number in series. This adaptation would be reasonable. The reason for this was described as follows: if the working range of each sarcomere becomes too large for reasons such as the aforementioned two conditions, the sarcomere should be elongated. When the working range of each sarcomere becomes larger than 3.65 μm, actin and myosin cannot interact (Gordon et al. 1966), in other words, muscle force cannot be produced. To avoid this problem, the sarcomere number in series would increase for preventing a very large elongation of each sarcomere that would lead to no interaction of actin and myosin. Therefore, an increase in sarcomere number in series (i.e. increase in fascicle length) should occur. However, these are special cases (i.e., not physiological conditions). During resistance training in humans, sarcomere should not work in such non-physiological working range. In addition, from a geometrical viewpoint, in a fusiform muscle such as biceps brachii and/or a pennate muscle with a small pennation angle, such as sartorius (Ward et al. 2009), it is difficult to elongate fascicle length to a large extent by resistance training because the origin and insertion does not change. Considering these, the fascicle length would not increase with resistance training substantially, at least, in physiological conditions.

The reason why we measured fascicle length at three joint angles is as follows: If we compare the fascicle length only at one specific joint angle between the resistance-trained and untrained individuals, and confirm no difference in length, there is still a possibility that fascicle length is not identical between the groups. Specifically, if the fascicle length-joint angle relationship differs between the groups, we cannot judge whether fascicle length is longer or not according to the data only obtained at one specific joint angle. Taken this into consideration, we obtained the fascicle length at three different joint angles including long and short fascicle length regions. As a result, no differences in fascicle length were observed at all joint angles. Therefore, we judged that the fascicle length did not differ between the resistance-trained and untrained individuals.

In this study, the pennation angle was larger in the training group than in the control group in both the VL and MG. These results were in agreement with previous studies, which reported the increase in pennation angle with resistance training (Aagaard et al. 2011; Blazevich and Giorgi 2001; Kawakami et al. 1995) and which reported that highly-trained athletes had larger pennation angle than untrained subjects (Kawakami et al. 1993). Joint torque has been considered to be attenuated by a pennation angle because as the pennation angle increases, the efficiency of force transmission decreases. Indeed, Ikegawa et al. (2008) reported that the negative correlation was found between the pennation angle and joint force. However, considering the extent of difference in pennation angle observed in the current study, the aforementioned attenuation effect would not be large. Specifically, the extent of difference in pennation angle observed in the current study was in the range of 3.6°–6.5° although we adopted highly-trained athletes and sedentary individuals. In the case of the 25° plantar flexion condition in MG [the largest difference in pennation angle was observed between training (30.3°) and control (23.8°) groups], the efficiency of force transmission is 91 and 86%, respectively. If the muscle force is 1,000 N, available force is 910 and 860 N, respectively. The difference is only 50 N (5% with respect to the 1,000 N). Taking this into account, the negative effect of the increased pennation angle that would occur in association with muscle hypertrophy would be small.

Previous studies comparing fascicle length among subjects reported that fascicle length was different between sprinters and non-sprinters (Abe et al. 2001), and between sprinters and distance runners (Abe et al. 2000); it was longer in sprinters. This may be caused by innate factors and not training plasticity. In sprinters, it is obvious that a larger shortening velocity of the muscle fibre is needed. As mentioned in the introduction, a long fascicle (i.e., large numbers of sarcomere in series) can shorten faster than a short fascicle (Bodine et al. 1982; Sacks and Roy 1982; Spector et al. 1980). Taken together, those with a naturally long fascicle lengths may become sprinters, because obtaining a long fascicle with resistance training would be difficult, considering the results of the present study.

This study was a cross-sectional study and not a longitudinal one. Thus, there is a possibility that our result might be caused by inter-individual differences. The subjects in highly-trained individuals would be well-endowed subjects because all the subjects in the training group had attended the national level sport festivals. Thus, the subjects in the training group would tend to have a longer fascicle length by nature because a longer fascicle length is advantageous from the view of shortening velocity (Bodine et al. 1982; Sacks and Roy 1982; Spector et al. 1980). Taken this into consideration, it is reasonable that highly-trained individuals have longer fascicle length compared to untrained individuals. However, our results does not support this idea. Thus, the possibility that genetic difference masks the influence of muscle size on the fascicle length (in other words, the control group had a longer fascicle than the training group in nature) would therefore, be low. In the future, longitudinal study adopting longer intervention period such as over 12 months, which is enough to evoke dramatic muscle hypertrophy is needed.

## Conclusions

We confirmed that although muscle size was significantly different between resistance-trained and untrained individuals, there was no difference in the fascicle length between them. These results indicate that fascicle length is not associated with muscle size, and suggest that fascicle length would not increase with resistance training, at least, in the physiological conditions. In other words, muscle hypertrophy is not necessarily accompanied with increase in fascicle length.
